# Pressure Sensitivity of SynGAP/PSD‐95 Condensates as a Model for Postsynaptic Densities and Its Biophysical and Neurological Ramifications

**DOI:** 10.1002/chem.201905269

**Published:** 2020-03-13

**Authors:** Hasan Cinar, Rosario Oliva, Yi‐Hsuan Lin, Xudong Chen, Mingjie Zhang, Hue Sun Chan, Roland Winter

**Affiliations:** ^1^ Physical Chemistry I—Biophysical Chemistry Faculty of Chemistry and Chemical Biology TU Dortmund Otto-Hahn-Strasse 4a 44227 Dortmund Germany; ^2^ Department of Biochemistry Faculty of Medicine University of Toronto Toronto Ontario M5S 1A8 Canada; ^3^ Molecular Medicine Hospital for Sick Children Toronto Ontario M5G 0A4 Canada; ^4^ Division of Life Science State Key Laboratory of Molecular Neuroscience Hong Kong University of Science and Technology Clear Water Bay Kowloon, Hong Kong China

**Keywords:** high pressure, liquid–liquid phase separation, protein condensates, SynGAP/PSD-95

## Abstract

Biomolecular condensates consisting of proteins and nucleic acids can serve critical biological functions, so that some condensates are referred as membraneless organelles. They can also be disease‐causing, if their assembly is misregulated. A major physicochemical basis of the formation of biomolecular condensates is liquid–liquid phase separation (LLPS). In general, LLPS depends on environmental variables, such as temperature and hydrostatic pressure. The effects of pressure on the LLPS of a binary SynGAP/PSD‐95 protein system mimicking postsynaptic densities, which are protein assemblies underneath the plasma membrane of excitatory synapses, were investigated. Quite unexpectedly, the model system LLPS is much more sensitive to pressure than the folded states of typical globular proteins. Phase‐separated droplets of SynGAP/PSD‐95 were found to dissolve into a homogeneous solution already at ten‐to‐hundred bar levels. The pressure sensitivity of SynGAP/PSD‐95 is seen here as a consequence of both pressure‐dependent multivalent interaction strength and void volume effects. Considering that organisms in the deep sea are under pressures up to about 1 kbar, this implies that deep‐sea organisms have to devise means to counteract this high pressure sensitivity of biomolecular condensates to avoid harm. Intriguingly, these findings may shed light on the biophysical underpinning of pressure‐related neurological disorders in terrestrial vertebrates.

## Graphical abstract

**High pressure sensitivity of postsynaptic densities**: Quite unexpectedly, a binary protein condensate mimicking postsynaptic densities, which are protein assemblies underneath the plasma membrane of excitatory synapses, reveals high pressure sensitivity, which may shed light on the biophysical underpinnings of pressure‐related neurological disorders.

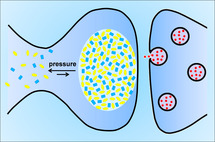

## Introduction

Biological cells need to orchestrate a large number of biochemical reactions in a spatiotemporally precise manner, which is facilitated by compartmentalization of cellular space. In addition to utilizing “classical” lipid bilayer membranes to achieve compartmentalization (e.g., plasma membrane, lysosomes, endoplasmic reticulum, mitochondria), membraneless compartments consisting of phase‐separated liquid‐like droplets have attracted significant attention in the last 10 years.^1^, ^2^, ^3^, ^4^, ^5^, ^6^, ^7^ Such membraneless compartments—generally referred to as biomolecular condensates—are ubiquitous and central to many cellular processes, including but not limited to cell growth, division, migration, and cell–cell communication.^1^, ^3^, ^8^, ^9^ Examples include various ribonucleoprotein‐enriched cytoplasmic granules, nucleoli, centrosomes, clusters of proteins involved in signaling, and postsynaptic densities.^6^, ^10^, ^11^ One advantage of such membraneless bodies over lipid‐bilayer‐bound compartments is that their biological function can be switched on and off more rapidly by regulating the liquid–liquid phase separation (LLPS) that underlies the formation and dissolution of the condensed droplet phase.

As LLPSs are generally dependent on temperature, pressure, and cosolutes, biomolecular condensates can contribute toward in vivo responses to environmental stress factors. Model LLPS systems in vitro have been demonstrated to respond to changes in temperature, pH and ionic strength.^5^, ^10^, ^12^ By comparison, high hydrostatic pressure (HHP) as a stress factor for biomolecular LLPS is much less explored.^7^, ^13^, ^14^, ^15^ Yet, a large fraction of the earth biosphere thrives under HHP, reaching pressures up to about 1 kbar (100 MPa, ≈1000 atm) in the deep sea and even beyond in the sub‐seafloor crust.^16^, ^17^ HHP studies on biomolecular condensates are thus necessary for understanding the physical basis of extant life in the deep sea, which might also be the birth place of life on earth.^16^ Aside from this direct relevance to deep‐sea biology, pressure serves as a useful physical probe of biomolecular interactions. As increasing pressure favors states with lower volumes, pressure‐dependence experiments can reveal low‐volume configurational states that are functionally important, but difficult to detect under ambient conditions.^18^, ^19^, ^20^, ^21^, ^22^, ^23^, ^24^, ^25^ Seeking progress in this context, we recently started investigating effects of pressure on the LLPS of simple one‐component protein systems, such as lysozyme, α‐elastin, γ‐crystallin, and the intrinsically disordered region of the DEAD‐box helicase Ddx4.^7^, ^13^, ^14^, ^15^ Here, we explore a more complex aqueous system consisting of two major proteins of the postsynaptic densities in neurons.

High hydrostatic pressure is known to affect various biomolecular systems, including lipid membranes, proteins such as enzymes, membrane transporters, the cytoskeleton, and nucleic acid hairpins.^18^, ^19^, ^22^, ^26^, ^27^, ^28^ Depending on the system, pressures of several hundred to thousand atmospheres are needed to induce significant conformational and, by inference, meaningful functional changes. Differently, exposure of vertebrates to high pressure results in severe neurological disorders known as high pressure neurological syndrome (HPNS),^29^ which starts to take place already at tens of atmospheres. They consist of altered electroencephalogram (EEG), dizziness, loss of coordination including tremor and convulsions.^30^, ^31^, ^32^, ^33^ Although this syndrome is among the most pressure‐sensitive processes known to date, its underlying physiological and biomolecular basis is still largely unknown. What is known so far only is that release of various neurotransmitters is suppressed and the function of some receptors and ion channels is perturbed.^30^, ^31^, ^32^, ^33^ The molecular effects of pressure on more complex synaptic assemblies, such as the postsynaptic densities, are still terra incognita.^33^

Synapses represent a unique type of membrane‐semi‐enclosed compartment that control signal transmission in all nervous systems. Underneath the postsynaptic plasma membranes of the synapse resides a protein‐rich sub‐compartment known as postsynaptic density (PSD), an assembly which is responsible for receiving, interpreting, and storage of signals transmitted by presynaptic axonal termini.^6^, ^10^, ^11^ PSDs have been shown to be composed of hundreds of densely packed proteins forming large assemblies with a few hundred nanometer in width and 30–50 nm in thickness.^34^, ^35^ Extensive studies have also revealed numerous protein–protein interactions that organize the PSD protein network.^6^, ^11^, ^36^ SynGAP and PSD‐95 are two very abundant proteins existing at a near stoichiometric ratio in PSD,^37^ and mutations of either of SynGAP or PSD‐95 are known to cause human psychiatric disorders, such as intellectual disorders (ID) and autism.^38^, ^39^, ^40^ SynGAP predominantly localizes in PSDs through specifically binding to PSD‐95.^41^, ^42^ SynGAP, a brain‐specific GTPase‐activating protein, forms a parallel coiled‐coil trimer capable of binding to multiple copies of PSD‐95. Importantly, this multivalent SynGAP/PSD‐95 interaction leads to the formation of liquid–liquid phase separation, both in vitro and in the living cell.^6^, ^10^, ^11^ The manner in which individual SynGAP and PSD‐95 monomers are associated to form higher‐order complexes in the LLPS state is not well understood in structural detail. The amino acid compositions of the part of the molecules known to be involved in the interaction suggest that the favorable contacts may involve a combination of hydrophobic and π‐related interactions.

To explore a possible role of LLPS in pressure‐induced neurological disorder, UV/Vis and fluorescence spectroscopy, turbidity measurements, light and fluorescence microscopy in various high‐pressure sample cells were used to study the structure and phase properties of the SynGAP/PSD‐95 system, covering a pressure range up to about 1500 bar. As shown below, LLPS of the SynGAP/PSD‐95 system is highly pressure sensitive and becomes unstable well below the 1 kbar range that can be encountered by organisms in the deep sea.

## Results

### Concentration and pressure dependence of SynGAP/PSD‐95 LLPS

SynGAP and PSD‐95 were prepared following previously described procedures.^6^ To prepare samples for the present experiments, SynGAP and PSD‐95 stock solutions were diluted to the desired concentration with Tris buffer and mixed in a ratio of 1:1. Liquid droplet formation upon entering the LLPS region was examined by monitoring the turbidity (apparent absorption) through light scattering at 400 nm using a UV/Vis spectrometer (Shimadzu UV‐1800). The temperature of the sample cell was controlled by an external water thermostat. Measurements were carried out at 25 °C and 37 °C. The pressure‐dependent measurements were carried out using a home‐built high‐pressure optical cell.^14^, ^15^ Sapphire with a diameter of 20 mm and a thickness of 10 mm was used as the window material. Pressure was applied by using a high‐pressure hand pump and was measured by a pressure sensor.

**Figure 1:**
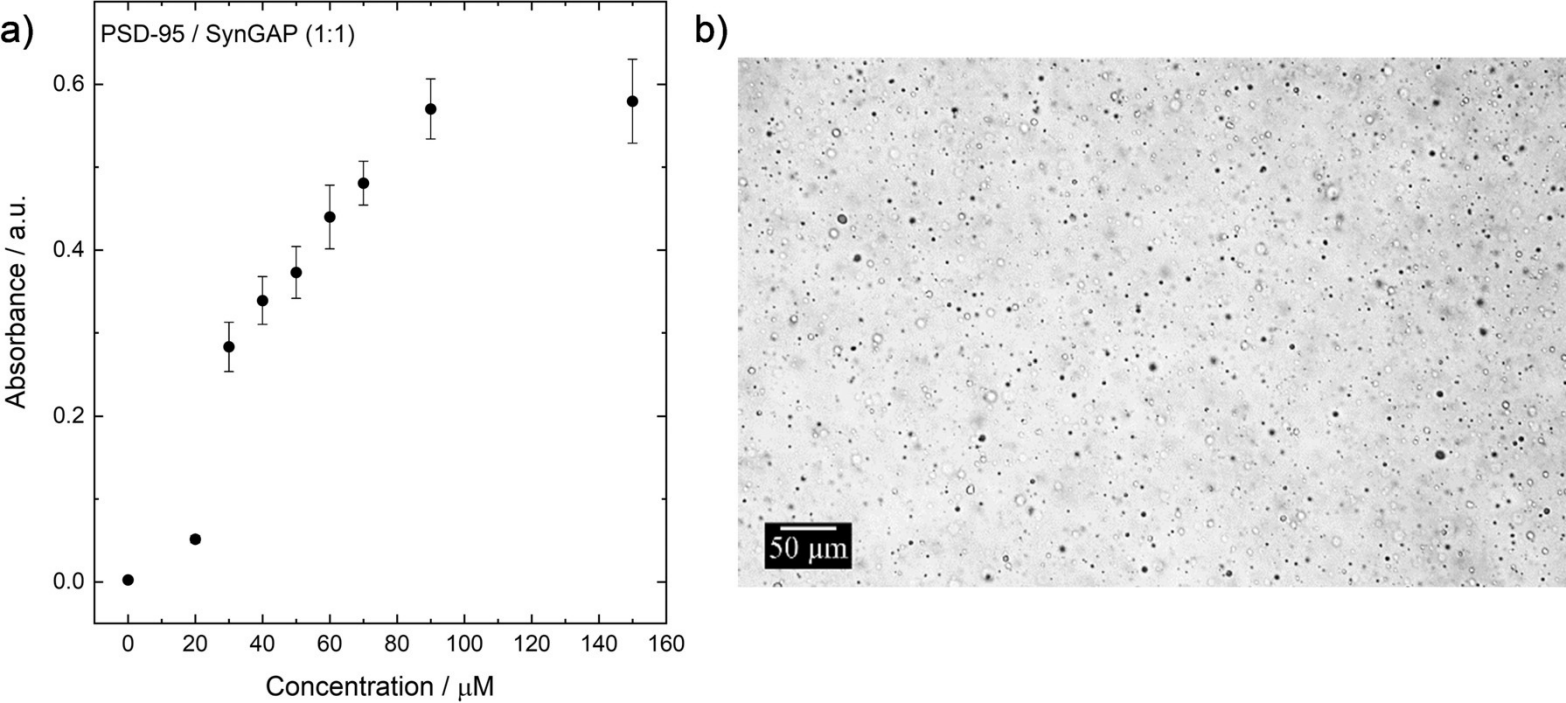
a) Concentration‐dependent turbidity measurements (at 400 nm) of SynGAP/PSD‐95 (1:1) solutions at *T*=25 °C, b) LLPS and droplet formation of a 50 μm SynGAP/PSD‐95 solution at *T*=25 °C.

To reveal the effect of protein concentration on the appearance of LLPS, we first studied the concentration dependence of the turbidity upon increasing the concentration of PSD95/SynGAP (1:1). As seen in Figure 1, we observe the expected increase in turbidity of the solution with increasing concentration of the protein mixture, indicating phase separation and droplet formation already at concentrations above 20 μm at 25 °C, in agreement with literature data.^6^ Beyond a protein concentration of 90 μm, the turbidity reaches a plateau value, which may indicate maximal droplet formation. However, the plateau is more likely caused by fusion of droplets and macroscopic phase separation at high protein concentrations, leading to an apparent plateau in light scattering.

**Figure 2:**
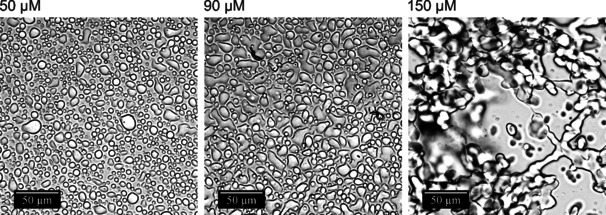
Light microscopy measurements of SynGAP/PSD‐95 solutions at different protein concentrations, ranging from 50 to 150 μm (*T*=25 °C).

To visualize the concentration‐dependent phase behavior of the SynGAP/PSD‐95 system, light microscopy studies were carried out. As depicted in Figure 1 b, immediately after mixing the two proteins, macroscopic phase droplets are formed in the solution, with droplet diameters up to about 5 μm. With time, droplet size increases. Within 15 min, macroscopic phase droplets are formed which sink to the bottom, forming extended liquid‐liquid phase separation regions on the bottom window surface of the microcopy cell. Figure 2 depicts the phase droplets at different protein concentrations when the objective focal point of the recorded images was on the inner window surface. The diameter of the condensed‐phase droplets increases with increasing protein concentration. At a concentration of 150 μm, a percolating network of the droplet phase has formed, which extends over 100 μm, a scenario which is in accordance with the results obtained by the turbidity measurements at high protein concentrations.

**Figure 3:**
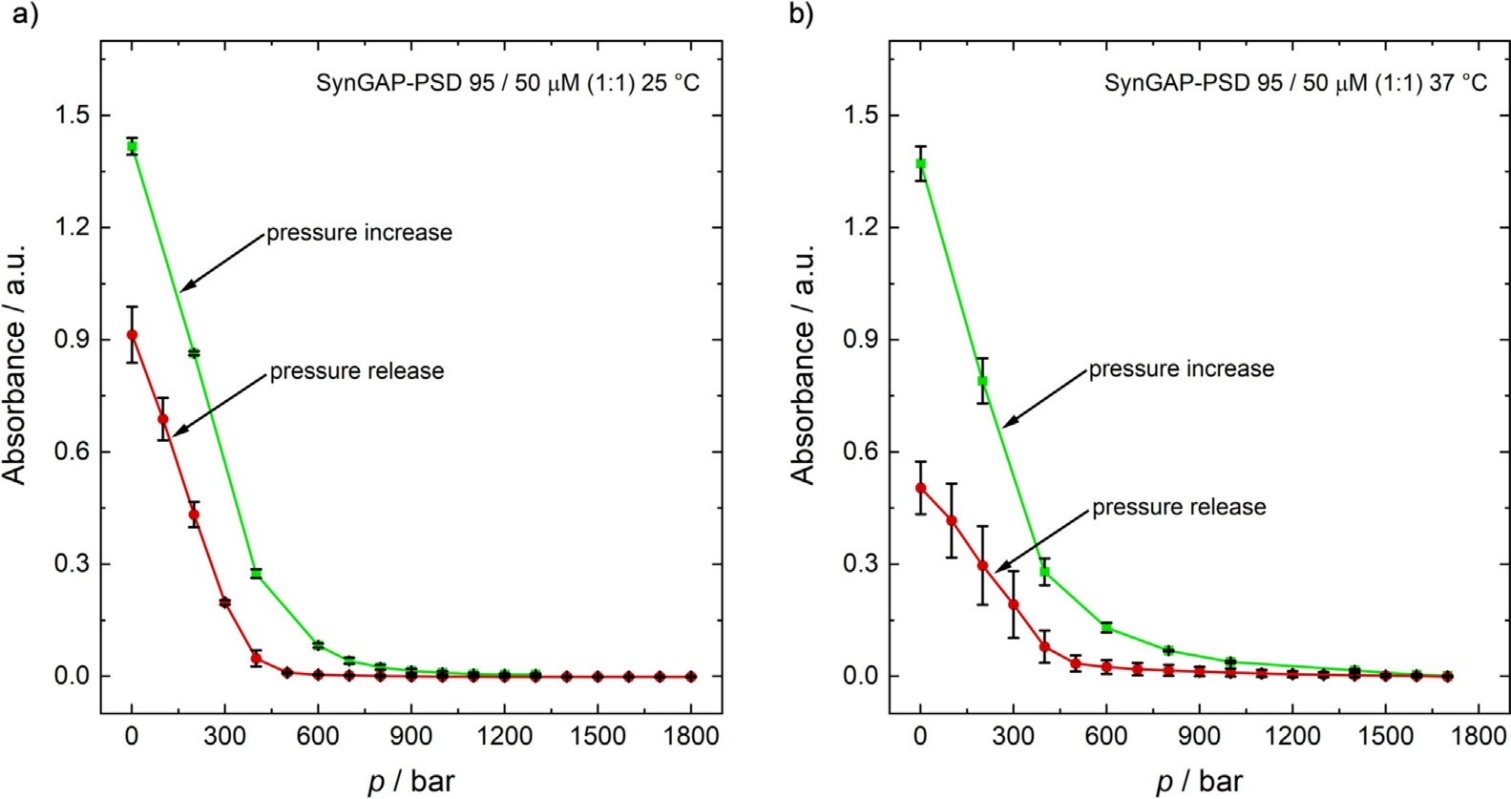
a) UV/Vis absorption spectra (400 nm) of a SynGAP/PSD‐95 (50 μm, 1:1) solution as a function of increasing and decreasing pressure at a) *T*=25 °C and b) *T*=37 °C. Error bars are given for at least three independent measurements. The stepwise increase or decrease of pressure was carried out using a manually operated pressure pump. The time to set the desired pressure was approx. 10–20 s until a constant pressure reading was obtained. If the sample is kept for a longer period of time (15–20 min), the small liquid droplets condense to larger phase droplets, leading to a slow decrease of the absorbance values.

Figure 3 shows the pressure‐dependent turbidity data of a 50 μm SynGAP/PSD‐95 (1:1) solution at two temperatures, 20 °C and 37 °C. 50 mm Tris solution (100 mm NaCl, 1 mm EDTA, 1 mm DTT) with a pH of 7.8 was used as buffer system. The pressure‐dependent UV/Vis data depicted in Figure 3 indicate that increasing the pressure beyond approximately 600 bar leads to a homogeneous phase at *T*=25 °C, the amount of droplets seems to decrease continuously up to that pressure, however. As can be seen in Figures 3 b, in the depressurization direction, the turbidity of the solution increases at about 400 bar, that is, the cloud point pressure is shifted to slightly lower pressures. In fact, a certain degree of hysteresis is expected for this type of nucleation‐induced phase transition. Several pressurization and depressurization cycles reveal that the process is apparently fully reversible.

In addition, pressure‐dependent turbidity investigations were carried out at 37 °C, which corresponds to the physiological temperature of humans. No drastic changes in the transition pressures are observed compared to the 25 °C data (Figure 3 b), the transition pressures seem to shift only to slightly higher values.

**Figure 4:**
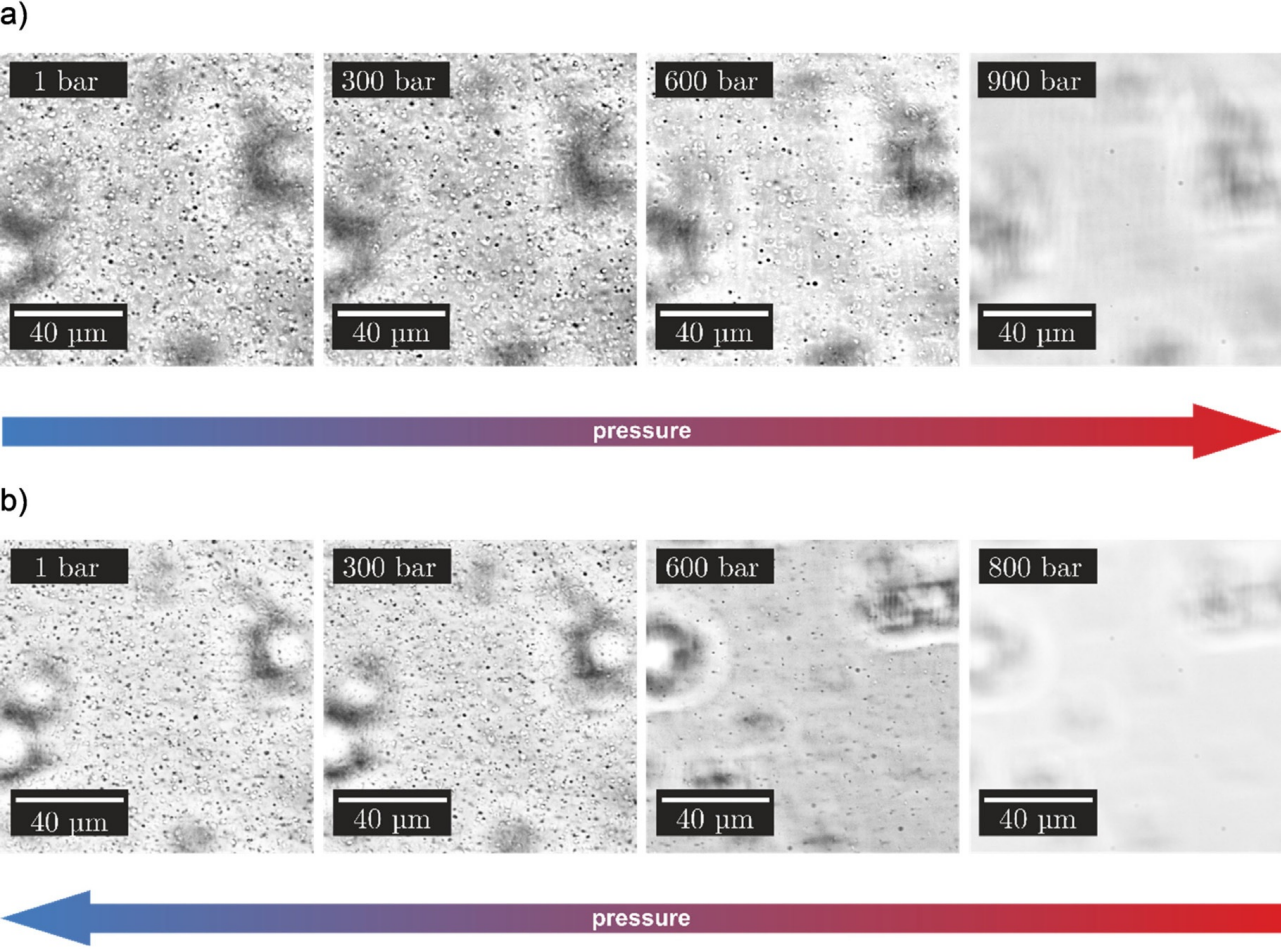
Representative light microscopy images of the SynGAP/PSD‐95 (1:1) solution (50 μm) at *T*=25 °C (bulk phase behavior), a) with increasing pressure from 1 to 600 bar, and b) with decreasing pressure from 800 bar to 1 bar (*T*=25 °C).

To validate the results obtained from the turbidity measurements, additional pressure‐dependent light microscopy measurements using a home‐built optical pressure‐cell with flat diamond windows were carried out, which operates up to about 1500 bar (see Figure S1, Supporting Information). All pressure‐dependent microscopy studies were performed with a 50 μm SynGAP/PSD‐95 (1:1) solution. Figure 4 shows selected microscopy images of the protein mixture at 25 °C, where the focus of the objective was adjusted to the inner part of the bulk solution, thereby avoiding surface effects on liquid droplet formation.

After mixing the two proteins, small phase droplets are immediately formed in the bulk solution with a diameter smaller than approximately 5 μm. With increasing pressure, the amount of phase droplets in the bulk solution decreases and a homogeneous single‐phase region was observed at a pressure still below 900 bar (Figure 4), in good agreement with the results obtained by the turbidity measurements. In the depressurization direction, the droplet formation could be detected at about 600 bar. For a better and more detailed visualization, we have added a movie (Movie 1 in the Supporting Information) showing images upon continuous pressure release of the sampe.

**Figure 5:**
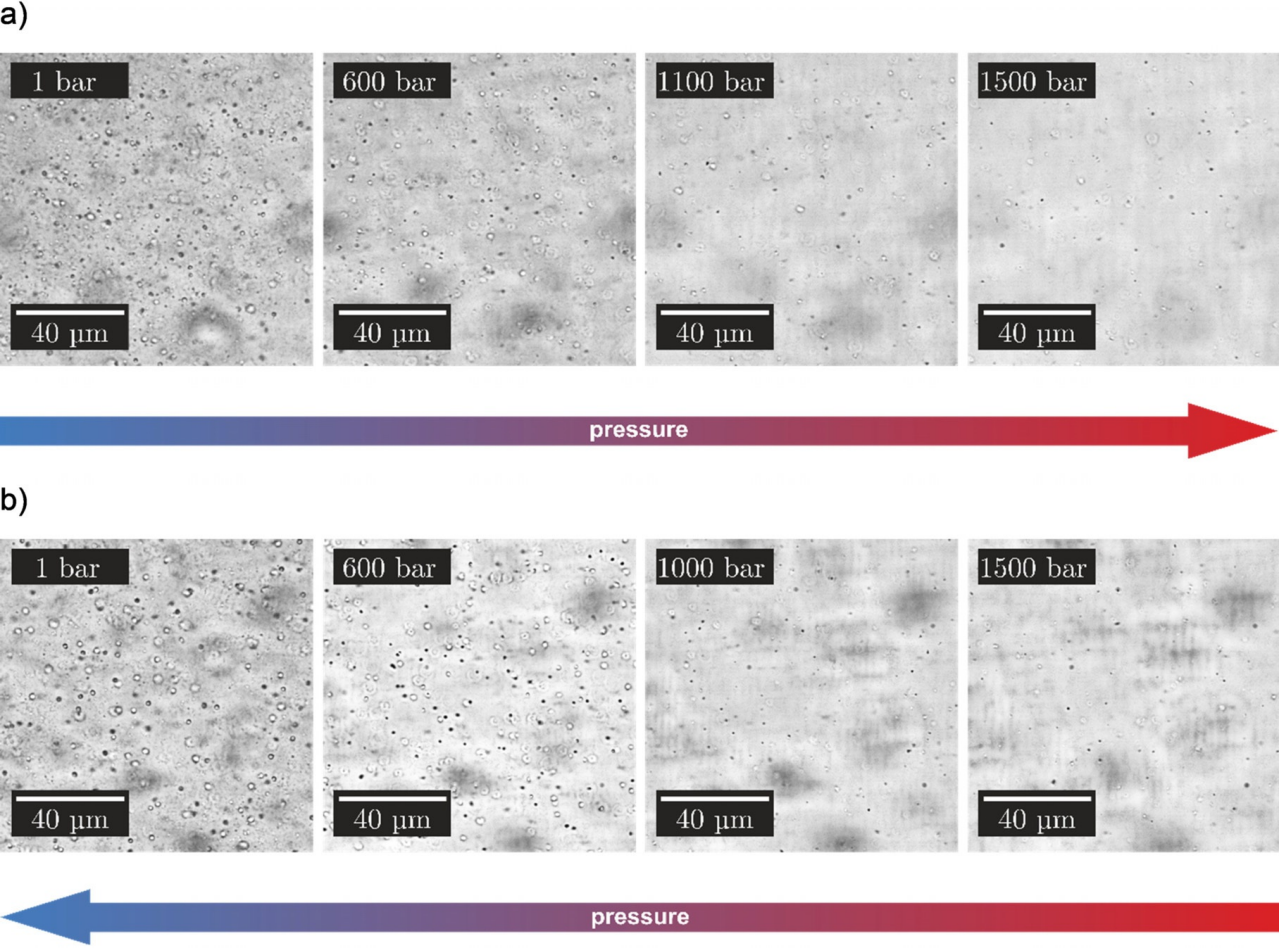
Representative light microscopy images of the SynGAP/PSD‐95 (1:1) solution (50 μm) at *T*=37 °C, a) with increasing pressure from 1 to 1500 bar, and b) with decreasing pressure from 1500 bar to 1 bar.

Figure 5 depicts the pressure dependence of the droplet formation of SynGAP/PSD‐95 at 37 °C. The measurements indicate that increasing the temperature to 37 °C shifts the transition pressure to higher values. In the entire pressure range covered (1–1500 bar), some phase droplets were always observed in the bulk, although their number decreases drastically with increasing pressure.

### Effect of pressure on SynGAP/PSD‐95 binding determined by FRET methodology

**Figure 6:**
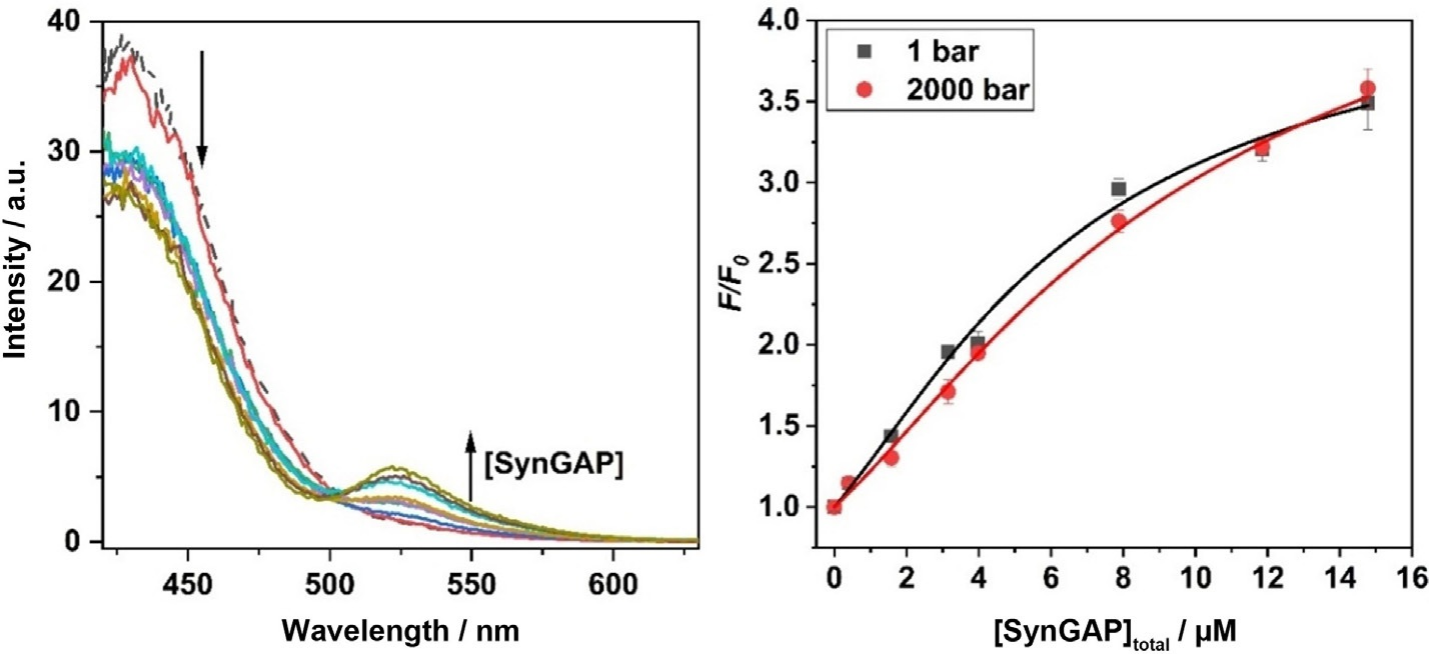
Fluorescence emission spectra (left) at ambient pressure and binding isotherms (right) obtained from the titration of Alexa 405‐labelled PSD‐95 with Alexa 488‐labeled SynGAP. The experiments were carried out at ambient temperature (25 °C) and five different pressures between 1 bar and 2000 bar (only data for 1 and 2000 bar are shown in this Figure). The curves in the right Figure are best fits to the experimental data according to a 1:1 binding site model.

Fluorescence measurements (Figure 6) were performed to determine the effect of hydrostatic pressure on the binding between SynGAP and PSD‐95. To this end, a series of solutions containing 2.5 μm of PSD‐95 labeled with Alexa 405 (donor) were prepared, and the concentration of SynGAP labeled with Alexa 488 (acceptor) was varied between 0–14.8 μm. The concentration of PSD‐95 was chosen such that the absorbance at the wavelength of excitation was less than 0.05 so as to avoid inner filter effects. The samples were then excited at 402 nm and the emission spectra were recorded in the range 420–630 nm by using a high‐pressure quartz cuvette with a path length of 0.4 cm. The spectra were collected at *T*=25 °C and at pressures of 1, 500, 1000, 1500 and 2000 bar. The extent of binding was evaluated by following the increase of fluorescence intensity at about 522 nm due to the Förster resonance energy transfer (FRET) between the Alex 405‐labeled PSD‐95 and the Alexa 488‐labeled SynGAP. The binding curves were obtained by plotting *F*/*F*_0_ as a function of total SynGAP concentration (in μm), in which *F*_0_ and *F* denote the fluorescence intensities at 522 nm in the absence and in the presence of SynGAP, respectively. The experimental data points were well fitted using a 1:1 binding site model.

The dissociation constant, *K*_d_, obtained from the data fitting procedure are reported in Table 1 (see also Figure S2 in the Supporting Information for all data). The results indicate that increasing pressure causes the dissociation constant for complex formation to increase slightly, that is, pressure disfavors the formation of the SynGAP/PSD‐95 complex. This trend is consistent with our data on pressure‐dependent LLPS; but the effect of pressure on the *K*_d_ is rather small. Hence, it is likely that other effects also play important roles in the marked pressure sensitivity of the LLPS of the SynGAP/PSD‐95 system, as we will discuss below.

**Table 1:** Pressure‐dependence of the dissociation constant, *K*_d_, of the SynGAP/PSD‐95 complex.

Pressure [bar]	*K*_d_ [μm]^[a]^
1	1.72±0.09
500	1.79±0.06
1000	2.08±0.09
1500	2.27±0.05
2000	2.17±0.09

[a] Errors are the standard deviations on curve fitting.

## Discussion

As stated above, hydrostatic pressure is one of the environmental constraints in our biosphere which has had substantial impact upon the evolution of a wide variety of aquatic organisms. Though the effect of pressure on simple biomolecular systems, such as lipid bilayers, proteins and nucleic acids, is quite well understood,^18^, ^19^, ^20^, ^21^, ^22^, ^23^, ^24^, ^25^, ^26^, ^27^, ^28^ the effect of HHP on more complex biomolecular assemblies is still largely unknown. In this study, we explored the effect of pressure on liquid‐phase droplets and the LLPS of two major components of PSDs. PSDs concentrates and organizes a multitude of proteins, serving as a signaling machinery in response to synaptic activities.^6^, ^10^, ^11^ Our results show the LLPS of the SynGAP/PSD‐95 model system for PSD is among the most pressure‐sensitive biomolecular assemblies identified so far. An increase of pressure of several ten‐to‐hundred bars can lead to a drastic decrease of phase‐separated droplets and the disappearance of the phase separation region at ambient temperature happens at around 600 bar.

From a biophysical perspective, it is particularly interesting that the pairwise interaction between SynGAP and PSD‐95 turned out to be rather pressure‐insensitive (Table 1), suggesting that the binding interface of a single SynGAP/PSD‐95 complex by itself is largely devoid of empty cavities and hence rather densely packed. Indeed, the pairwise complex is pressure stable up to the 2 kbar range. Considering that the pressure dependence of pairwise binding is insufficient to account for the pressure sensitivity of SynGAP/PSD‐95 droplets, a plausible physical rationalization is that a significant larger void (cavity) volume inaccessible to water molecules is associated with the multiple‐molecule interaction network in the condensed phase than the dilute phase of SynGAP/PSD‐95. In general, void volume can arise geometrically from imperfect packing in compact conformational states,^13^ as in the folded structures of globular proteins.^21^, ^43^, ^44^, ^45^ Void‐volume effects can offer a rationalization for pressure‐dependent LLPS of biomolecular condensates as well, wherein the voids are envisioned to be transient whereas the voids in folded proteins are essentially static.^7^, ^14^

**Figure 7:**
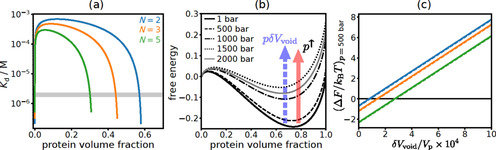
Rudimentary estimation of the increase in void/cavity volume associated with the formation of the condensed SynGAP/PSD‐95 phase based on a Semenov–Rubinstein‐type gelation model.^47^ (a) Phase diagrams (coexistence curves) of three alternate formulations in which *N* is an effective number of rigid‐body units and, hence, a larger *N* assumes more flexible individual SynGAP and PSD‐95 molecules. The grey band marks the range of pairwise dissociation constants in Table 1. (b) Pressure‐dependent free energy profiles of the *N*=2 model. The vertical variable is *F*_V_(*φ*,*p*)−*φF*_V_(1,1) as defined in the Supporting Information, in which *φ*=protein volume fraction, *F*_V_ is free energy per unit volume in units of *k*_B_ *T*, *k*_B_ is Boltzmann constant and *T*=300 K is absolute temperature. It must be noted that phase separation is not affected^48^ by any term linear in *φ*. The pink arrow highlights destabilization of the *φ* ≈0.7 condensed‐phase local minimum as pressure increases (*p* ^↑^); the purple dashed arrow indicates that a higher void volume can destabilize the condensed phase. (c) Δ*F* is the difference in free energy, per protein complex, between the low‐*φ* local maximum and the high‐*φ* local minimum [corresponding, for example, to the free energy difference between *φ*≈0.1 and *φ*≈0.7 for the *N*=2 case in (b)] in the presence of a hypothetical δ*V*_void_. Results are reported for *p*=500 bar for the three models in (a) using the same color code for *N*. Dashed lines are obtained using a slightly varied definition of the local free energy minima and maxima, as described in the Supporting Information.

We explore void‐volume effects in SynGAP/PSD‐95 droplets semi‐quantitatively by using an extremely simple model of SynGAP/PSD‐95 phase separation. Given that the interactions between SynGAP and PSD‐95 are structurally specific,^6^, ^46^ it is more appropriate to use a gelation‐type model that entails a specific number of “stickers” for each molecule^47^ rather than a Flory–Huggins polymer model with nonspecific contact interactions,^48^ even though the SynGAP/PSD‐95 droplets are liquid‐like rather than gel‐like. Since both SynGAP (1308 amino acid residues) and PSD‐95 (721 residues) are largely folded and tend to form complexes with a 3:2 stoichiometry,^6^ our model considers a single generic molecular species with limited structural flexibility and a molecular volume *V*_p_ which equals to 5000 times that of an amino acid residue (≈139.6 Å^3^).^49^ Based on the SynGAP/PSD‐95 interaction pattern,^46^ each of these generic units in our model is assigned four stickers and the pressure‐dependent interaction strengths between a pair of stickers are taken to be those given in Table 1. Details of this model, which by itself neglects void‐volume effects, are provided in the Supporting Information. Results of our analysis are shown in Figure 7.

As surmised, Figure 7 a shows that the pressure‐dependent *K*_d_ values in Table 1 afford only a very limited variation in LLPS propensity (narrow grey band). They do not account for the fact that LLPS is not observed experimentally at about 500 bar and higher (the entire grey band is in the phase‐separated regime). This situation is indicated again by the model free‐energy profiles in Figure 7 b, which are all bimodal with a favored condensed phase. Recognizing that the model does not address void‐volume effects, we consider how an auxiliary increase in void volume, δ*V*_void_, from the dilute to condensed phase would affect phase behaviors. At pressure *p*, δ*V*_void_ raises the condensed‐phase free energy by *p*δ*V*_void_ relative to that of the dilute phase (dashed arrow in Figure 7 b). Hence, a positive δ*V*_void_ is expected to destabilize condensed droplets. Because phase boundaries are governed by second derivatives of free energy with respect to volume fraction,^50^ an exact determination of void‐volume effects on phase behaviors would require knowledge of void volume for all protein volume fractions (not merely the difference between the condensed and dilute phases). Nonetheless, rough estimates based only on δ*V*_void_ are possible because, if the free energy of the condensed phase is raised above the barrier between the dilute and condensed phases, it is likely that phase separation would no longer occur. By using such an approximate procedure and requiring that no LLPS occurs at 500 bar, δ*V*_void_ as a fraction of protein molecular volume is estimated to be 0.01–0.03 % (Δ*F*=0 intercepts in Figure 7 c). Notably, these values are not affected significantly by varying the model parameters *N* (up to *N*=10) and *V*_p_ (e.g., decreasing *V*_p_ to that of 2000 residues as for a pairwise SynGAP/PSD‐95 complex). In this regard, we also note that if the *K*_d_ values in Table 1 were reduced (which is possible because the ITC‐measured *K*_d_ value for *p*=1 in Figure 3 B of Ref. 6 is about an order of magnitude smaller), a proportionally larger δ*V*_void_ would be estimated. Despite modeling as well as experimental uncertainties noted and taking all the above considerations together, we deem it likely that void volumes play a key role in the pressure sensitivity of the SynGAP/PSD‐95 droplets. In this perspective, the condensed droplet phase becomes unstable under high pressure, in accordance with Le Châtelier's principle,^28^ partly because a reduction of void volume is achieved upon dissolution of the droplets and formation of a homogeneous dilute phase, which is also favored by a higher mixing entropy.^7^

The present estimate of δ*V*_void_/*V*_p_≈0.01–0.03 % is physically plausible as it does not entail creation of large water‐inaccessible voids that would be difficult to maintain in the liquid state. In fact, this δ*V*_void_/*V*_p_ ratio is far smaller than the δ*V*_void_/*V*_p_≈7 % estimated for folded globular proteins.^45^ It would appear, therefore, that the pressure sensitivity of SynGAP/PSD‐95 droplets arises not from a large δ*V*_void_. Rather, it is likely a consequence of the combined impact of a modest δ*V*_void_ and a set of droplet‐forming cohesive interactions (Table 1), which are much weaker than the interactions favoring the folded states of globular proteins.

## Conclusion

As mentioned above, proper assembly of PSDs are critical to neuron function. An intriguing case in point is that down‐scaling of PSDs can be induced by sleep in mice.^51^ Mutations and dysfunction of PSDs are linked to human neuropsychiatric and neurodevelopmental disorders.^29^, ^30^, ^31^, ^32^, ^52^ Considering that the nervous system is one of the most sensitive targets of high pressure,^29^, ^33^ it is tantalizing to find that the phase‐transition pressure of the PSD‐mimicking SynGAP/PSD‐95 system is about an order of magnitude smaller compared to those typically leading to protein unfolding.^18^, ^28^ Although much further effort, such as construction of reconstituted PSDs using more complex in vitro systems,^11^ will be needed to elucidate the structure–function relation of PSDs, the present observations offer a novel approach to investigate neurological effects of hydrostatic pressure. If the pressure sensitivity of natural PSDs are similar or even higher than that of the SynGAP/PSD‐95 droplets, our findings may help decipher the underlying mechanisms of neurological disorders of vertebrates under pressures that are not much higher than atmospheric pressure at sea level,^33^ including onset of high‐pressure neurological syndrome at approximately 10 bar.^29^

## Conflict of interest

The authors declare no conflict of interest.

## Supporting information

As a service to our authors and readers, this journal provides supporting information supplied by the authors. Such materials are peer reviewed and may be re‐organized for online delivery, but are not copy‐edited or typeset. Technical support issues arising from supporting information (other than missing files) should be addressed to the authors.

Supplementary

Supplementary

## References

[1] C. P. Brangwynne , P. Tompa , R. V. Pappu , Nat. Phys. 2015, 11, 899–904.

[2] D. Zwicker , R. Seyboldt , C. A. Weber , A. A. Hyman , F. Jülicher , Nat. Phys. 2017, 13, 408–413.

[3] S. F. Banani , H. O. Lee , A. A. Hyman , M. K. Rosen , Nat. Rev. Mol. Cell Biol. 2017, 18, 285–298. 28225081 10.1038/nrm.2017.7 PMC7434221

[4] C. D. Keating , Acc. Chem. Res. 2012, 45, 2114–2124. 22330132 10.1021/ar200294y PMC3525015

[5] J. P. Brady , P. J. Farber , A. Sekhar , Y. H. Lin , R. Huang , A. Bah , T. J. Nott , H. S. Chan , A. J. Baldwin , J. D. Forman-Kay , L. E. Kay , Proc. Natl. Acad. Sci. USA 2017, 114, E8194–E8203. 28894006 10.1073/pnas.1706197114 PMC5625912

[6] M. Zeng , Y. Shang , Y. Araki , T. Guo , R. L. Huganir , M. Zhang , Cell 2016, 166, 1163–1175. 27565345 10.1016/j.cell.2016.07.008 PMC5564291

[7] H. Cinar , Z. Fetahaj , S. Cinar , R. M. Vernon , H. S. Chan , R. Winter , Chem. Eur. J. 2019, 25, 13049–13069. 31237369 10.1002/chem.201902210

[8] A. A. Hyman , C. A. Weber , F. Jülicher , Annu. Rev. Cell Dev. Biol. 2014, 30, 39–58. 25288112 10.1146/annurev-cellbio-100913-013325

[9] J. D. O'Connell , A. Zhao , A. D. Ellington , E. M. Marcotte , Annu. Rev. Cell Dev. Biol. 2012, 28, 89–111. 23057741 10.1146/annurev-cellbio-101011-155841 PMC4089986

[10] Z. Feng , M. Zeng , X. Chen , M. Zhang , Biochemistry 2018, 57, 2530–2539. 29648450 10.1021/acs.biochem.8b00313

[11] M. Zeng , X. Chen , D. Guan , J. Xu , H. Wu , P. Tong , M. Zhang , Cell 2018, 174, 1172–1187. 30078712 10.1016/j.cell.2018.06.047

[12] J. A. Riback , C. D. Katanski , J. L. Kear-Scott , E. V. Pilipenko , A. E. Rojek , T. R. Sosnick , D. A. Drummond , Cell 2017, 168, 1028–1040. 28283059 10.1016/j.cell.2017.02.027 PMC5401687

[13] K. Julius , J. Weine , M. Berghaus , N. König , M. Gao , J. Latarius , M. Paulus , M. A. Schroer , M. Tolan , R. Winter , Phys. Rev. Lett. 2018, 121, 038101. 30085800 10.1103/PhysRevLett.121.038101

[14] H. Cinar , S. Cinar , H. S. Chan , R. Winter , Chem. Eur. J. 2018, 24, 8286–8291. 29738068 10.1002/chem.201801643

[15] H. Cinar , S. Cinar , H. S. Chan , R. Winter , J. Am. Chem. Soc. 2019, 141, 7347–7354. 30985120 10.1021/jacs.8b13636

[16] I. Daniel , P. Oger , R. Winter , Chem. Soc. Rev. 2006, 35, 858–875. 17003893 10.1039/b517766a

[17] F. Meersman , I. Daniel , D. Bartlett , R. Winter , R. Hazael , P. F. McMillan , Rev. Mineral. Geochem. 2013, 75, 607–648.

[18] High-Pressure Bioscience: Basic Concepts, Applications and Frontiers (Eds.: K. Akasaka, H. Matsuki), Springer, Amsterdam, 2015.

[19] J. L. Silva , A. C. Oliveira , T. C. R. G. Vieira , G. A. P. de Oliveira , M. C. Suarez , D. Foguel , Chem. Rev. 2014, 114, 7239–7267. 24884274 10.1021/cr400204z

[20] R. Mishra , R. Winter , Angew. Chem. Int. Ed. 2008, 47, 6518–6521; 10.1002/anie.200802027 18646033

[21] J. Roche , J. A. Caro , D. R. Norberto , P. Barthe , C. Roumestand , J. L. Schlessman , A. E. Garcia , B. E. García-Moreno , C. A. Royer , Proc. Natl. Acad. Sci. USA 2012, 109, 6945–6950. 22496593 10.1073/pnas.1200915109 PMC3344970

[22] K. Heremans , L. Smeller , Biochim. Biophys. Acta 1998, 1386, 353–370. 9733996 10.1016/s0167-4838(98)00102-2

[23] H. Y. Fan , Y. L. Shek , A. Amiri , D. N. Dubins , H. Heerklotz , R. B. , Jr. , Macgregor , T. V. Chalikian , J. Am. Chem. Soc. 2011, 133, 4518–4526. 21370889 10.1021/ja110495c

[24] H. R. Kalbitzer , Subcell. Biochem. 2015, 72, 179–197. 26174382 10.1007/978-94-017-9918-8_9

[25] S. Kapoor , G. Triola , I. R. Vetter , M. Erlkamp , H. Waldmann , R. Winter , Proc. Natl. Acad. Sci. USA 2012, 109, 460–465. 22203965 10.1073/pnas.1110553109 PMC3258604

[26] R. Winter , C. Jeworrek , Soft Matter 2009, 5, 3157–3173.

[27] R. Winter in High-Pressure Bioscience. Subcellular Biochemistry (Eds.: K. Akasaka, H. Matsuki), Springer, Amsterdam, 2015, pp. 345–370.

[28] R. Winter , Annu. Rev. Biophys. 2019, 48, 441–463. 30943042 10.1146/annurev-biophys-052118-115601

[29] A. E. Talpalar , Rev. Neurol. 2007, 45, 631–636. 18008270

[30] A. G. MacDonald , Philos. Trans. R. Soc. London Ser. B 1984, 304, 47–68. 6142479 10.1098/rstb.1984.0008

[31] Current Perspectives in High-Pressure Biology (Eds.: H. W. Jannasch, R. E. Marquis, A. M. Zimmermann), Academic Press, Cambridge, 1987.

[32] Basic and Applied High Pressure Biology (Eds.: P. B. Bennett, R. E. Marquis), University of Rochester Press, Rochester, 1994.

[33] Comparative High-Pressure Biology (Ed.: P. Sébert), Science Publishers, Enfield, 2010.

[34] X. Chen , C. Winters , R. Azzam , X. Li , J. A. Galbraith , R. D. Leapman , T. S. Reese , Proc. Natl. Acad. Sci. USA 2008, 105, 4453–4458. 18326622 10.1073/pnas.0800897105 PMC2393784

[35] K. M. Harris , R. J. Weinberg , Cold Spring Harbor Perspect. Biol. 2012, 4, a005587. 10.1101/cshperspect.a005587 PMC3331701 22357909

[36] J. Zhu , Y. Shang , C. Xia , W. Wang , W. Wen , M. Zhang , EMBO J. 2011, 30, 4986–4997. 22117215 10.1038/emboj.2011.428 PMC3243629

[37] D. Cheng , C. C. Hoogenraad , J. Rush , E. Ramm , M. A. Schlager , D. M. Duong , P. Xu , S. R. Wijayawardana , J. Hanfelt , T. Nakagawa , M. Sheng , J. M. Peng , Mol. Cell. Proteomics 2006, 5, 1158–1170. 16507876 10.1074/mcp.D500009-MCP200

[38] M. H. Berryer , F. F. Hamdan , L. L. Klitten , R. S. Møller , L. Carmant , J. Schwartzentruber , L. Patry , S. Dobrzeniecka , D. Rochefort , M. Neugnot-Cerioli , J.-C. Lacaille , Z. Niu , C. M. Eng , Y. Yang , S. Palardy , C. Belhumeur , G. A. Rouleau , N. Tommerup , L. Immken , M. H. Beauchamp , G. S. Patel , J. Majewski , M. A. Tarnopolsky , K. Scheffzek , H. Hjalgrim , J. L. Michaud , G. Di Cristo , Hum. Mutat. 2013, 34, 385–394. 23161826 10.1002/humu.22248

[39] F. F. Hamdan , J. Gauthier , D. Spiegelman , A. Noreau , Y. Yang , S. Pellerin , S. Dobrzeniecka , M. Cote , E. Perreau-Linck , L. Carmant , G. D′Anjou , É. Fombonne , A. M. Addington , J. L. Rapoport , L. E. Delisi , M.-O. Krebs , F. Mouaffak , R. Joober , L. Mottron , P. Drapeau , C. Marineau , R. G. Lafrenière , J. C. Lacaille , G. A. Rouleau , J. L. Michaud , N. Engl. J. Med. 2009, 360, 599–605. 19196676 10.1056/NEJMoa0805392 PMC2925262

[40] M. J. Parker , A. E. Fryer , D. J. Shears , K. L. Lachlan , S. A. McKee , A. C. Magee , S. Mohammed , P. C. Vasudevan , S.-M. Park , V. Benoit , Am. J. Med. Genet. A 2015, 167, 2231–2237. 10.1002/ajmg.a.37189 PMC4744742 26079862

[41] H.-J. Chen , M. Rojas-Soto , A. Oguni , M. B. Kennedy , Neuron 1998, 20, 895–904. 9620694 10.1016/s0896-6273(00)80471-7

[42] J. H. Kim , D. Liao , L.-F. Lau , R. L. Huganir , Neuron 1998, 20, 683–691. 9581761 10.1016/s0896-6273(00)81008-9

[43] C. L. Dias , H. S. Chan , J. Phys. Chem. B 2014, 118, 7488–7509. 24933471 10.1021/jp501935f

[44] H. Krobath , T. Chen , H. S. Chan , Biochemistry 2016, 55, 6269–6281. 27775315 10.1021/acs.biochem.6b00802

[45] C. R. Chen , G. I. Makhatadze , Nat. Commun. 2017, 8, 14561. 28169271 10.1038/ncomms14561 PMC5309723

[46] M. Zeng , F. Ye , J. Xu , M. Zhang , J. Mol. Biol. 2018, 430, 69–86. 29138001 10.1016/j.jmb.2017.11.003

[47] A. N. Semenov , M. Rubinstein , Macromolecules 1998, 31, 1373–1385.

[48] Y.-H. Lin , J. D. Forman-Kay , H. S. Chan , Biochemistry 2018, 57, 2499–2508. 29509422 10.1021/acs.biochem.8b00058

[49] S. Miyazawa , R. L. Jernigan , Macromolecules 1985, 18, 534–552.

[50] Y.-H. Lin , J. Song , J. D. Forman-Kay , H. S. Chan , J. Mol. Liq. 2017, 228, 176–193.

[51] L. de Vivo , M. Bellesi , W. Marshall , E. A. Bushong , M. H. Ellisman , G. Tononi , C. Cirelli , Science 2017, 355, 507–510. 28154076 10.1126/science.aah5982 PMC5313037

[52] T. Kaizuka , T. Takumi , J. Biochem. 2018, 163, 447–455. 29415158 10.1093/jb/mvy022

