# Supplementary material for: Pressure Sensitivity of SynGAP/PSD‐95 Condensates as a Model for Postsynaptic Densities and Its Biophysical and Neurological Ramifications
Source: Chemistry. 2020 Mar 13;26(48):11024–31. doi: 10.1002/chem.201905269 (PMC7496680; doi:10.1002/chem.201905269)
Supplement: Supplementary file 1 — Supplementary [file CHEM-26-11024-s001.pdf]

Chemistry–A European Journal

Supporting Information

Pressure Sensitivity of SynGAP/PSD-95 Condensates as a Model for Postsynaptic Densities and Its Biophysical and Neurological Ramifications

Hasan Cinar,[a] Rosario Oliva,[a] Yi-Hsuan Lin,[b, c] Xudong Chen,[d] Mingjie Zhang,[d] Hue Sun Chan,*[b] and Roland Winter*[a]

Additional Figures and Movies

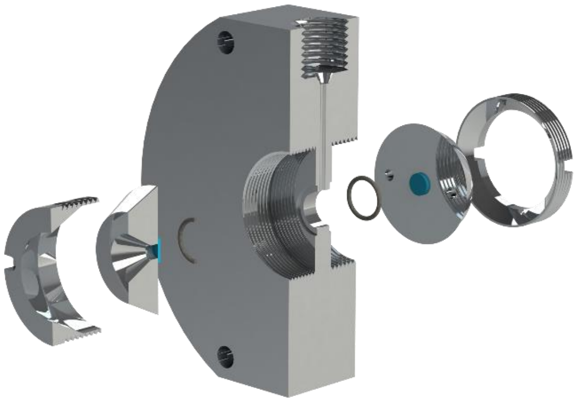

Figure SI 1. Schematic drawing of the home-built high-pressure cell for microscopy imaging. Pressure was generated hydrostatically by a high-pressure hand pump with water as pressure-transmitting fluid. Flat diamond windows were used as optical window material on both sides. The design fits to a standard inverted microscope with off-the-shelf long-distance microscope objectives.

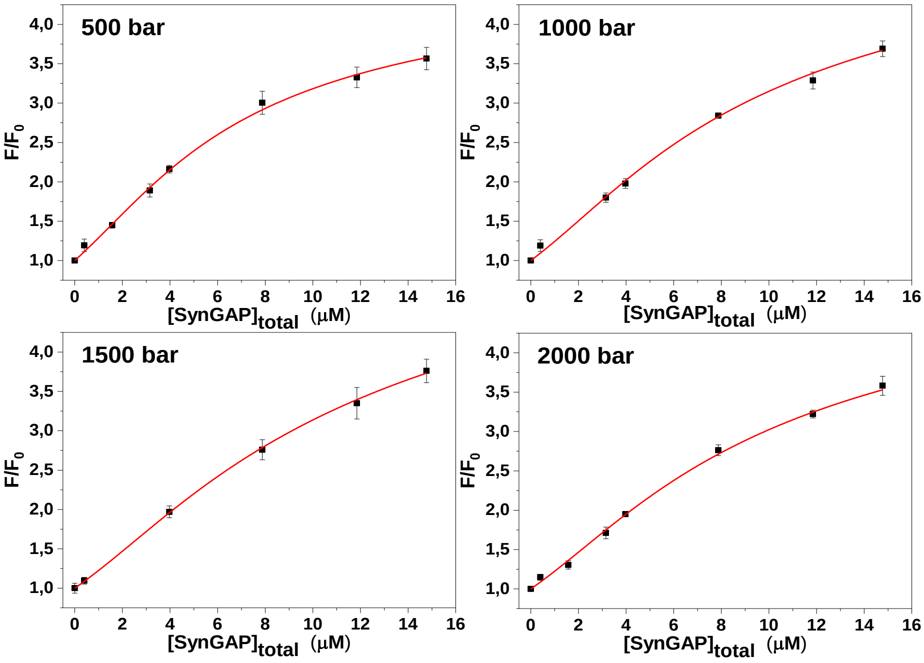

Figure SI 2. Binding isotherms obtained from the titration of PSD 95 labeled with Alexa 405 with SynGAP labeled with Alexa 488 at the hydrostatic pressures reported. The temperature was set at 25 °C.

Movie 1. Light microscopy data in the bulk phase of 90 μM human SynGAP/PSD-95 system (50 mM TRIS, 150 mM NaCl, pH 7.4) during pressure release at T = 25 °C.

Details of a Gelation-Type Model for SynGAP/PSD-95 phase separation

As discussed in the main-text, because of the structural specificity of SynGAP/PSD-95 binding,[S1] we construct a Semenov-Rubinstein-type gelation model[S2] with the following considerations: (i) For simplicity, the only polymer species in the model is a generic SynGAP/PSD-95 complex, which may be loosely identified with the one with a 3:2 stoichiometry that has been deduced experimentally.[S3] (ii) Because individual SynGAP and PSD-95 molecules are largely folded, the polymers in the model are taken to possess limited structural flexibility. Accordingly, each polymer consists of only a small number, N, of monomer units, with each monomer unit representing an essentially rigid section of the generic complex consisting of many amino acid residues. (iii) Let $\mathcal{V}$ be the volume of such a monomer unit in the model and $V_{\rm p}$ be the volume of the polymer representing the generic SynGAP/PSD-95 complex, it follows that $\mathcal{V} = V_{\rm p}/N$. (iv) Each polymer has $f = 4$ "stickers", which correspond roughly to the two PDZ3 and two GK domains[S1] in a 3:2 SynGAP/PSD-95 complex. For simplicity, we do not distinguish between these two different domains and treat all stickers equally. Under these assumptions, the free energy per unit volume in units of $k_{\rm B}T$, where $k_{\rm B}$ is Boltzmann constant and T is absolute temperature, is given by[S2]

$$F_{\mathcal{V}}(\phi,p) = (\phi/N)\ln(\phi) + (1-\phi)\ln(1-\phi) + (\phi f/N)\{r(\phi,p)/2 + \ln[1-r(\phi,p)]\} ,$$

where ϕ is protein (polymer) volume fraction, p is pressure in units of bar, and $r(\phi,p)$ is the fraction of stickers that form pairs. Following ref. S2,

$$r(\phi,p) = 1 - \{[4\phi f\lambda(p)/N + 1]^{1/2} - 1\}/[2\phi f\lambda(p)/N] ,$$

where $\lambda(p) = 1/[K_{\rm d}(p)\mathcal{V}]$ is the Boltzmann factor for the formation of a sticker pair, with $K_{\rm d}(p)$ being the pressure-dependent dissociation constants in Table 1 of the main text. The phase diagrams (coexistence curves) in Figure 7a of the main-text were obtained from the $F_{\mathcal{V}}(\phi,p)$ expression above using standard methods.[S4] By definition, the free energy F per polymer (protein complex) at pressure p is given by $F(\phi)|_p = Nk_{\rm B}T\, F_{\mathcal{V}}(\phi,p)/\phi$. In addition to the free energy profiles $F_{\mathcal{V}}(\phi,p) - \phi F_{\mathcal{V}}(1,1) = F_{\mathcal{V}}(\phi,p) + 8.552\phi$ used in Figure 7b of the main-text (which applies the same subtraction $\phi F_{\mathcal{V}}(1,1)$ to all p's), we also considered $F_{\mathcal{V}}(\phi)|_p - \phi F_{\mathcal{V}}(1)|_p - (1-\phi)F_{\mathcal{V}}(0)|_p$ (which has all p-dependent pure-phase contributions subtracted) and used the local minima and maxima along these alternate free energy profiles to obtain the slightly different estimates of $\delta V_{\rm void}/V_{\rm p}$ provided by the dashed lines in Figure 7c of the main-text.

S1. M. Zeng, F. Ye, J. Xu, M. Zhang, PDZ ligand binding-induced conformational coupling of the PDZ-SH3-GK tandems in PSD-95 family MAGUKs. *J. Mol. Biol*. **430**, 69-86 (2018).

S2. A. N. Semenov, M. Rubinstein, Thermoreversible gelation in solutions of associative polymers. 1. Statics. *Macromolecules* **31**, 1373-1385 (1998).

S3. M. Zeng, Y. Shang, Y. Araki, T. Guo, R. L. Huganir, M. Zhang, Phase transition in postsynaptic densities underlies formation of synaptic complexes and synaptic plasticity. *Cell* **166**, 1163–1175 (2016).

S4. Y.-H. Lin, J. Song, J. D. Forman-Kay, H. S. Chan, Random-phase-approximation theory for sequence-dependent, biologically functional liquid-liquid phase separation of intrinsically disordered proteins. *J. Mol. Liq*. **228**, 176-193 (2017).
